# Development of Antibodies to Ustekinumab Is Associated with Loss of Response in Patients with Inflammatory Bowel Disease

**DOI:** 10.3390/jcm12103395

**Published:** 2023-05-10

**Authors:** Xavier Roblin, Gérard Duru, Konstantinos Papamichael, Adam S. Cheifetz, Sandy Kwiatek, Anne-Emmanuelle Berger, Mathilde Barrau, Louis Waeckel, Stephane Nancey, Stephane Paul

**Affiliations:** 1 Department of Gastroenterology, University Hospital of Saint-Etienne, F42270 Saint-Etienne, France; 2 CIRI—Centre International de Recherche en Infectiologie, Team GIMAP, Université Claude Bernard Lyon 1, Inserm, U1111, CNRS, UMR5308, F42023 Saint-Etienne, Francestephane.paul@chu-st-etienne.fr (S.P.); 3 CIC 1408 Inserm Vaccinology, University Hospital of Saint-Etienne, F42055 Saint-Etienne, France; 4 Department of Statistics, University Claude Bernard Lyon 1, F69008 Lyon, France; 5 Center for Inflammatory Bowel Disease, Beth Israel Deaconess Medical Center, Harvard Medical School, Boston, MA 02215, USA; 6 Department of Gastroenterology, University Hospital of Saint-Denis de la Réunion, F97400 Saint-Denis de la Réunion, France; 7 Immunology Department, iBioThera Reference Center, University Hospital of Saint-Etienne, F42055 Saint-Etienne, France; 8 Department of Gastroenterology, Lyon Sud Hospital, Hospices Civils de Lyon, University Claude Bernard Lyon 1 and INSERM U1111, F69003 Lyon, France

**Keywords:** ustekinumab, immunogenicity, Crohn’s disease, drug-tolerant assay

## Abstract

Monitoring of anti-drug antibodies in patients on ustekinumab is not routinely recommended in patients with inflammatory bowel disease (IBD) due to low rates of immunogenicity. Aim of study: The purpose of this study was to investigate the relationship between anti-drug antibodies detected by a drug-tolerant assay and loss of response (LOR) to therapy in a cohort of patients with IBD being treated with ustekinumab. Patients and Methods: This retrospective study consecutively enrolled all adult patients with moderate to severe active IBD who had at least 2 years of follow-up after ustekinumab was initiated. LOR was defined as CDAI > 220 or HBI > 4 for Crohn’s disease (CD) and partial Mayo subscore > 3 for ulcerative colitis (UC) and with a modification in disease management. Results: Ninety patients were included (78 CD and 12 UC; mean age 37 years). Median levels of anti-ustekinumab antibodies (ATU) were significantly higher in patients with LOR compared to those with ongoing clinical response (15.2 µg/mL-eq CI (7.9–21.5) and 4.7 µg/mL-eq CI (2.1–10.5), respectively; *p* = 0.04). The area under the ROC curve (AUROC) for ATU in predicting LOR was 0.76. The optimal cut-off point for identifying patients with LOR was 9.5 µg/mL-eq with a sensitivity of 80% and specificity of 85%. Uni- and multivariate analyses showed that serum ATU ≥ 9.5 µg/mL-eq (hazard ratio (HR) 2.54, 95%CI (1.80–5.93)), *p* = 0.022, prior vedolizumab (HR 2.78, 95%CI (1.09–3.34), *p* = 0.019) and prior azathioprine (HR 0.54, 95%CI (0.20–0.76), *p* = 0.014) exposures were the only factors independently associated with LOR to UST. Conclusion: In our real-life cohort, ATU was identified as an independent predictor of LOR to ustekinumab in patients with IBD.

## 1. Introduction

The current therapeutic landscape for moderate to severe inflammatory bowel disease (IBD) is rapidly evolving. There are novel biologics now available, such as vedolizumab and ustekinumab. Despite the expanding therapeutic armamentarium for patients with IBD, anti-tumor necrosis factor (TNF) therapies remain widely utilized in routine practice. In some countries, anti-TNF use is mandatory as a first line therapy in specific situations based on reimbursement policies. However, not all patients respond to induction therapy with anti-TNF and 20% to 40% of patients who initially respond may lose response over time. The formation of anti-drug antibodies is one of the leading contributors to loss of response (LOR) for anti-TNF therapy; although, immunogenicity can also occur with the more novel biologics [1,2]. Ustekinumab (UST), a fully human immunoglobulin IgG1k monoclonal antibody directed against the common p40 subunit of IL-12 and IL-23, has proven to be effective and safe for the treatment of Crohn’s disease (CD) and ulcerative colitis (UC) [3,4,5]. In addition, the relationship between UST exposure and CD outcomes has been investigated in a post hoc analysis of the pivotal UNITI trials [6]. Similar to anti-TNF, serum concentrations of UST are associated with efficacy outcomes during both induction and the maintenance therapy. CD patients with serum UST concentrations in the higher quartiles were more likely to achieve a clinical and endoscopic remission during maintenance therapy. More recently, a prospective observational study reported that early UST concentrations (as early as one hour after iv drug administration) may help physicians to identify CD patients most likely to respond to therapy and those requiring dose-intensification [7]. In UC, an exposure–response relationship with UST concentrations has also been reported [8]. In the pivotal phase 3 trials of both CD and UC, immunogenicity of UST, assessed by a drug-tolerant assay, was low (<5%). Furthermore, most of the antibodies to UST (ATU) were transient and non-neutralizing [8]. Currently, the routine monitoring of ATU is not performed and may not be clinically relevant. However, patients with ATU may have lower median serum UST concentration compared with those without ATU resulting in lower clinical response [8]. Nevertheless, data on ATU and their relationship to disease outcomes in IBD are still scarce. We aimed to investigate the relationship between ATU detected by a drug-tolerant assay and LOR in a cohort of patients with IBD treated with UST.

## 2. Patients and Methods

### 2.1. Study Design, Disease Outcomes and Patient Characteristics

This was a retrospective study from two French tertiary referral IBD centers. The study included adult patients diagnosed with moderate to severe IBD based on standard clinical, endoscopic, histopathological and radiological criteria. All eligible patients received a single iv induction with ~6 mg/kg UST (260 mg for body weight ≤ 55 kg, 390 mg for body weight 55–85 kg, 520 mg for body weight > 85 kg) followed by 90 mg sc injections every 8 weeks. Eligible patients must be followed for at least 2 years in case of sustained clinical response under UST. Enrolled patients had at least one serum UST concentration and ATU. Patients with primary non-response to UST (defined by a lack of clinical response both at week 8 and 16) or patients who experienced a serious adverse event requiring withdrawal of therapy were excluded from the study. Patients were also excluded if they were less than 18 years old, received another induction and maintenance UST regimens, had an ostomy, exclusive perineal CD, restorative proctocolectomy with ileal pouch-anal anastomosis or had a positive rheumatoid factor. The latter were excluded to avoid any interference with the detection of ATU.

Loss of clinical response was defined as CDAI > 220 or HBI > 4 for CD and partial Mayo subscore > 3 for UC associated with the decision to modify disease management or treatment (dose intensification, add on steroids, swap into another treatment, IBD-related hospitalization or surgery) [9]. The study was performed in accordance with the Declaration of Helsinki, Good Clinical Practice and applicable regulatory requirements. Written informed consent was obtained from all patients participating in the study. All patients have given written consent also to the Biobank of St-Etienne. The study was approved by the French ethical committee, so-called Comité de Protection des Personnes (Number: 1849323).

### 2.2. Therapeutic Drug Monitoring

Ustekinumab samples were obtained at the end of follow-up (generally 2 years after starting ustekinumab) in patients without LOR or at the time of LOR. UST drug concentrations were measured using Theradiag iTrack10 (France). ATU were measured using a drug-tolerant immunoassay (all samples analyzed at St-Etienne), which was adapted and developed by using anti-human lambda chain as previously described [10,11,12,13,14]. Briefly, ustekinumab was coated on MaxiSorp ELISA plates in coating buffer at 10 µg/mL o/n at 4 °C. After, washing plates were saturated with PBS BSA 4%. Serial dilution in PBS BSA 1% was then used. Detection of ATU was performed using a goat F(ab’)2 anti-human lambda chain-HRP (Sigma-Aldrich, St. Louis, MO, USA). The identification of anti-ustekinumab antibodies with our drug-tolerant assay was performed twice in a blinded fashion. Blood donors (×50) were used to define a threshold value of ATU at 11 µg/mL-eq. Wild samples from the French blood bank were measured using the drug-tolerant assay to define a threshold value, adding two standard deviations.

### 2.3. Statistical Analysis

Continuous data were expressed as means ± standard deviations (SD) or medians with interquartile range (IQR), whereas nominal and ordinal data were expressed as numbers and percentages. Categorical data were compared using the Chi-square test or the Fischer’s exact test, as appropriate. Continuous data were compared using the Mann–Whitney tests and Wilcoxon’s matched-pair signed-rank test, as appropriate. A correlation between ATU or UST concentration and LOR during follow-up was analyzed using a reason test. Receiver operating characteristic (ROC) curves were plotted to compare the ability of continuous variables (i.e., UST concentrations and levels of ATU) to predict LOR and to identify optimal threshold values based on the Youden index. Variables associated with UST immunogenicity were assessed by uni- and multivariate analyses using COX regression analysis. All analyses were two-tailed, and *p* values <0.05 were considered statistically significant. Statistical analysis was performed using the SPSS statistical software (SPSS Inc., v26, Chicago, IL, USA).

## 3. Results

### 3.1. Patient’s Characteristics

**Figure 1:**
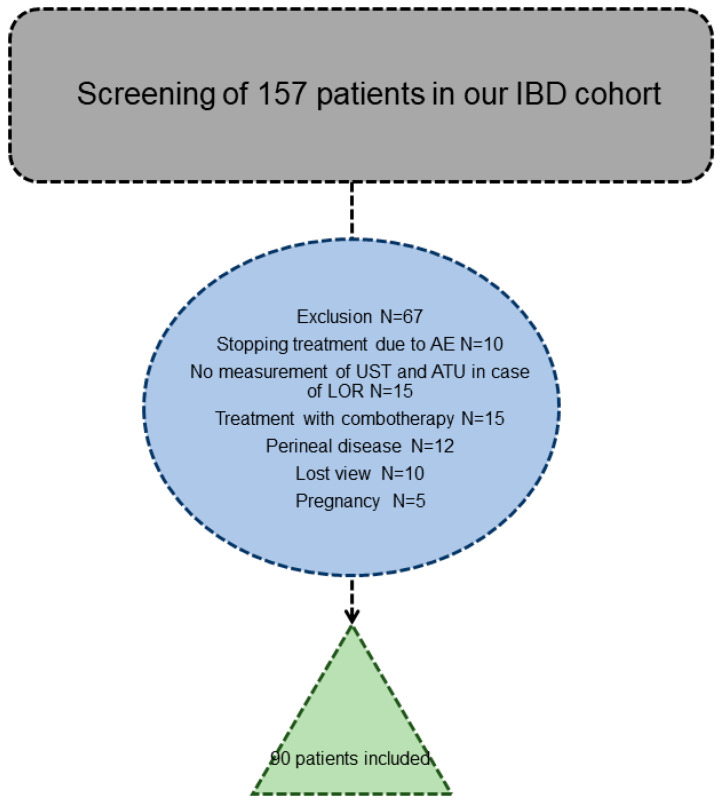
Synopsis of the study.

One hundred and fifty-seven patients with IBD on ustekinumab were identified, and after applying the exclusion criteria, 90 patients were included in the study (78 with CD and 12 with UC) (Figure 1 and Table 1).

The mean age was 37 ± 4 years, and the mean disease duration was 8.5 ± 3.5 years. In compliance with the recommendations of the French Health Authority, 88 patients were previously treated with anti-TNF therapy. Thirty patients had also received vedolizumab. Patient characteristics were similar between patients with and without LOR during follow-up except for prior exposure to vedolizumab and prior exposure to azathioprine (Table 1). Patients were followed for a median of 1.8 years (IQR: 0.9–2.8). Thirty-six patients had LOR on UST maintenance therapy (90 mg every 8 weeks), all of whom were dose-intensified to 90 mg every 4 weeks. A total of 12 out of the 36 optimized patients (33%) regained clinical remission (defined as CDAI < 150 or HBI < 4 for CD and partial Mayo subscore < 3 for UC) upon drug intensification.

### 3.2. Therapeutic Drug Monitoring

**Figure 2:**
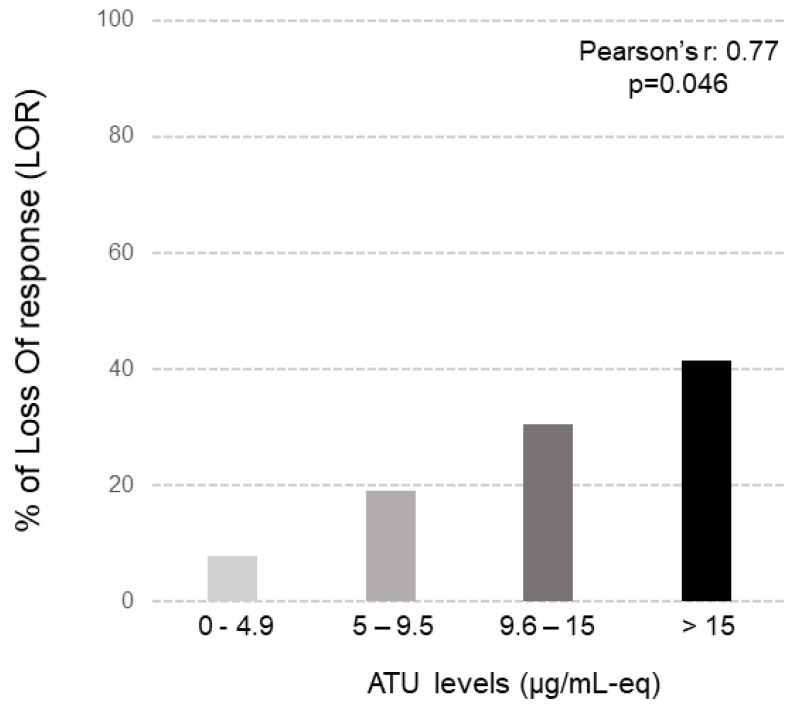
Correlation between ATU (antibodies to ustekinumab using drug-tolerant assay) levels and percentage of LOR in our IBD cohort.

Median serum UST concentrations did not differ between patients with or without LOR (4.21 IQR (2.02–5.20) µg/mL vs. 4.56 IQR (2.95–6.67) µg/mL, respectively, *p* = 0.570). Conversely, the median levels of ATU were significantly higher in patients with LOR compared to patients without LOR (15.2 IQR (7.9–21.5) µg/mL-eq vs. 4.7 IQR (2.1–10.5) µg/mL-eq, respectively; *p* = 0.04). ATU were positive in 30% of patients (42% for patients with LOR vs. 6% for patients without LOR; *p* = 0.045). There was a significant correlation between the levels of ATU and LOR during the follow-up (Pearson’s, r = 0.77, *p* = 0.046) (Figure 2).

**Figure 3:**
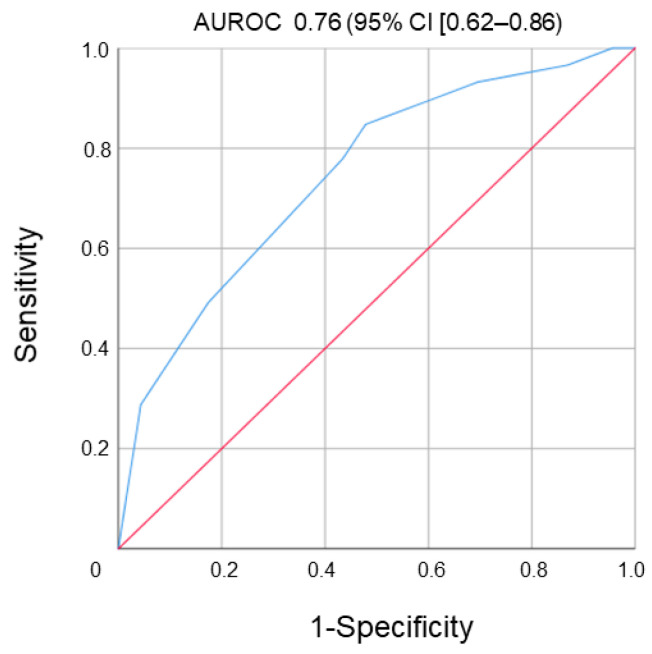
ROC curve to isolate threshold value of ATU to predict LOR during the follow-up.

The area under the ROC curve (AUROC) for ATU for predicting LOR was 0.76 (95% confidence interval (CI) (0.62–0.86), *p* = 0.035) (Figure 3).

The optimal cut-off to identify patients with LOR was 9.5 µg/mL-eq with a sensitivity of 80%, specificity of 85%, positive predictive value of 87% and a negative predictive value of 80%. There was no significant correlation between circulating UST concentrations and levels of ATU (Pearson’s, r = −0.093, *p* = 0.68). The median levels of ATU were equivalent in CD compared to UC patients (8.4 µg/mL-eq vs. 8.7 µg/mL-eq, respectively; *p* = 0.86).

Uni- and multivariate analysis demonstrated that serum ATU ≥ 9.5 µg/mL-eq (hazard ratio (HR) 2.54, 95%CI (1.80–5.93)), *p* = 0.022, prior vedolizumab (HR 2.78, 95%CI (1.09–3.34), *p* = 0.019) and prior azathioprine used alone (HR 0.54, 95%CI (0.20–0.76), *p* = 0.014) exposures were the only factors independently associated with LOR to UST (Table 2).

Patients with ATU levels exceeding the threshold of 9.5 µg/mL-eq, those who received prior vedolizumab therapy and those with no prior azathioprine therapy were more likely to have a SLR to UST.

## 4. Discussion

This is the first study to report that the presence of ATU > 9.5 μg/mL, detected by a drug-tolerant immunoassay, is an independent factor associated with LOR to UST. In contrast to the pivotal phase 3 trials and long-term extensions [8,15] where neutralizing ATU were detected in less than 5% of patients, in our cohort, ATU were detected in 30% of patients and the levels of ATU exceeded 9.5 µg/mL in 17% of IBD patients. Our findings also differ from previous studies in which ATU were not associated with LOR. These discrepancies may be explained by the distinct methods of ATU detection, the difference between the study population and the absence of concomitant immunosuppressants in our cohort. We also think that due to ustekinumab monotherapy without IS drug, we have an increased number of patients with anti-drug antibodies. In psoriatic arthritis, the authors showed that the rate of immunization was two times more under monotherapy ustekinumab than under a combination of treatment with methotrexate [16]. Moreover, generally, antibodies measurement in all studies were performed using a qualitative value conversely to our study using, for a first time, a quantitative assay. Moreover, all our patients were in failure to at least two anti-TNF drugs with probably in more than 20% of cases an immunogenic mechanism.

In addition, almost all the patients in our study were previously exposed to at least one anti-TNF agent (and almost half were exposed to two or more anti-TNFs) and/or to vedolizumab. Moreover, all phase 3 trial analyzing UST used a chemiluminescence assay [9,11]. The study of anti-drug antibodies by chemiluminescence assay is less clear with heterogeneous data. This technique also does not allow the measurement of ATU in the presence of drugs (drug-tolerant) without any dissociation step. Across the phase 3 trials, serum ustekinumab concentrations were dose-proportional and showed a positive association with clinical remission at week 8 (UNITI-1 and UNITI-2) and week 24 (IM-UNITI). At the end of induction (week 8), median ustekinumab concentrations were 2.1 and 6.4 µg/mL for the 130 mg and 6 mg/kg dose groups, respectively. Additionally, the median steady-state trough serum ustekinumab concentrations over time in the q8w group (1.97–2.24 µg/mL) were 3-fold higher than in the q12w group (0.61–0.76 µg/mL). In a real-world analysis of 59 patients from McGill University, a serum ustekinumab level of 4.5 µg/mL was associated with endoscopic response (72.2% sensitivity, 83.3% specificity; *p* = 0.0006; area under curve, 0.782). This level (compared to lower levels) was also associated with a composite outcome of steroid-free clinical remission and endoscopic response (50% vs. 15%, *p* = 0.024). Additionally, a ustekinumab level of 5 µg/mL, compared to lower levels, was associated with normal serum CRP (63.6% vs. 33%, *p* = 0.024). Conversely to our results, immunogenicity was low in these trials. In the CERTIFI trial, only 3 of the 427 Crohn’s disease patients with samples for analysis (0.7%) showed antibodies to ustekinumab through week 36. Likewise, the incidence of antibodies to ustekinumab was low (0.2%) in the UNITI studies across both dose groups. In IM-UNITI, the incidence of antibodies to ustekinumab through week 44 was similarly low (27/1154 patients, 2.3%). In a real-world experience at McGill University, none of the 49 patients tested had detectable antibodies to ustekinumab at 6 months [15].

Moreover, in a recent study, the authors analyzed the efficacy and safety of ustekinumab in pediatric patients with psoriasis. They reported in their cohorts than 10% of patients developed anti-drug antibodies [16]. In another study analyzing ustekinumab in adult patients with active psoriatic arthritis (PsA), a total of 615 adult patients with active PsA were randomized to placebo, ustekinumab 45 mg or ustekinumab 90 mg, at weeks 0, 4 and every 12 weeks through week 88 (last dose). Through week 108, a total of 49 of 591 patients (8.3%) tested positive for antibodies to ustekinumab. The incidence of antibodies to ustekinumab was similar between the two doses but was lower among patients receiving concomitant MTX (*n* = 13 of 287, 4.5%) compared with those not receiving MTX (*n* = 36 of 304, 11.8%). Patients who tested positive for antibodies to ustekinumab had lower mean serum ustekinumab concentrations than patients who had tested negative [17].

In the phase 3 pivotal trials, the higher quartiles of UST concentrations were positively correlated with favorable therapeutic outcomes in both CD and UC [6,8]. Interestingly, our findings failed to detect a relationship between UST concentrations and LOR. Likewise, we failed to detect any relationship between drug concentrations and ATU. This could be partially explained by the fact that UST concentrations were not only measured at trough (just prior to the injection) but also in between injections, as blood samples were taken at the discretion of the treating physician. In addition to the levels of ATU above the cut-off point of 9.5 µg/mL, we identified two other variables independently associated with LOR to UST. The first variable was the lack of previous use of azathioprine alone. It is possible that these patients had less a severe disease and are less likely to experience LOR to UST. Previous use of azathioprine was associated as protective in our study against LOR under UST (HR 0.54, 95%CI [0.20–0.76], *p* = 0.014). However, a metanalysis investigating the role of combination therapy found that combining UST with an immunomodulator is no more effective than monotherapy [18,19]. The second variable independently associated with LOR to UST was previous use of vedolizumab. In a large, retrospective, multi-center study of 1113 patients with CD aiming to identify predictors of response to UST, previous biologic exposure (including anti-TNF agents and vedolizumab) was associated with a greater risk for endoscopic failure [20]. On multivariable analysis, prior antitumor necrosis factor (hazard ratio, 0.72; 95% confidence interval, 0.49–0.99) and vedolizumab exposure (hazard ratio, 0.65; 95% confidence interval, 0.48–0.88) were independently associated with lower likelihoods of achieving endoscopic remission. Even if speculative, previous exposure to numerous biologics, such as anti-TNFs and vedolizumab, probably suggest more refractory patients with IBD who are more likely to fail subsequent UST. We acknowledge some limitations of our study, including the retrospective design with its potential biases. In addition, our definition of LOR did not include objective markers of inflammation, the definition of LOR without objective markers of inflammation, the small population sample size and no consecutive measurement for all patients treated with ustekinumab of anti-drug antibodies. There was also a rather small sample size. It would also be interesting to test the relationship between the HLA DQA1*05 allele and ATU, as it was previously reported that IBD patients carrying this allele were more likely to develop immunogenicity to anti-TNF therapy. The study did have several strengths in including the real-life cohort of patients, the use of a drug-tolerant assay, the long follow-up of the patients and the fact that ATU and UST measurements were blinded to disease clinical outcomes decreasing potential bias. In conclusion, using a drug-tolerant immunoassay in our cohort, ATU was be detected in a substantial proportion of patients with IBD and ATU > 9.5 µg/mL-eq was an independent predictor of LOR. These findings, if confirmed in larger and prospective studies, may facilitate decision making and help optimize management of patients losing response to UST.

## Figures and Tables

**Table 1:** Patients’ characteristics.

Patient Characteristics	Total	No LOR	LOR	*p*
Sex Ratio male/Female	1.1	1.0	1.1	0.870
Age (years) Mean ± SD	38.7 ± 6.3	37.4 ± 4.1	39.5 ± 7.2	0.760
Disease duration (years) median, (IQR)	8.5 (4.2–11.3)	7.7 (3.9–10.6)	9.9 (5.2–10.5)	0.450
Active smoking, *n* (%)	54 (60)	36 (66)	18 (50)	0.320
IBD type, *n* (%)				0.660
CD	78 (86)	48 (88)	30 (83)	
UC	12 (14)	6 (12)	6 (17)	
CD behavior, *n* (%)				0.320
B1	24 (38)	16 (33)	8 (26)	
B2	18 (22)	12 (25)	6 (20)	
B3	36 (40)	20 (41)	16 (54)	
CD (location %)				0.23
L1	32 (41)	20 (41)	12 (40)	
L2	11 (14)	9 (19)	2 (7)	
L3	35 (45)	19 (40)	16 (53)	
UC extension, *n* (%)				0.830
E1	2 (17)	1 (17)	1 (17)	
E2	6 (50)	4 (66)	2 (34)	
E3	4 (33)	1 (17)	3 (49)	
CDAI at inclusion, median, (IQR)	280 (240–330)	270 (245–325)	285 (250–340)	0.540
Full Mayo score, *n*	8 (8–9)	8 (7–8)	8 (8–9)	0.890
Previous treatments, *n* (%)				
-at least 1 anti-TNF	79 (80)	49 (90)	30 (83)	0.210
-2 anti-TNFs	36 (40)	21 (38)	15 (41)	0.670
-Vedolizumab	27 (30)	11 (20)	16 (45)	0.040
-AZA	30 (33)	21 (39)	9 (25)	0.030

IQR: interquartile range; CDAI: Crohn’s disease activity index; LOR: loss of response; AZA: azathioprine; TNF: tumor necrosis factor; IBD: inflammatory bowel disease; CD: Crohn’s disease; UC: ulcerative colitis.

**Table 2:** Factors associated with loss of response to ustekinumab.

	Univariate Analysis		Multivariate Analysis	
	*p*	HR (95% CI)	*p*	HR (95% CI)
Gender (male vs. female)	0.870	0.95 (0.29–4.02)		
Age	0.760	0.88 (0.56–3.55)		
Ustekinumab trough concentrations <4.5 µg/mL	0.450	1.18 (0.74–4.23)		
ATU ≥ 9.5 μg/mL-eq	0.045	2.81 (1.35–4.91)	0.022	2.54 (1.80–5.93)
UC vs. CD	0.300	1.40 (0.74–2.65)		
Prior vedolizumab therapy	0.060	1.56 (0.98–1.39)	0.019	2.78 (1.09–3.34)
Prior azathioprine therapy	0.028	0.66 (0.52–0.82)	0.014	0.54 (0.20–0.76)
Active smoking	0.650	1.12 (0.40–3.55)		
Duration of disease	0.450	0.67 (0.26–9.21)		
Prior use of two anti-TNFs	0.670	1.12 (0.32–8.72)		

HR: hazard ratio; ATU: antibodies to ustekinumab; CD: Crohn’s disease; UC: ulcerative colitis; TNF: tumor necrosis factor.

## Data Availability

Not applicable.

## Abbreviations

ATU	antibodies to ustekinumab
CD	Crohn’s disease
CRP	C-reactive protein
LOR	loss of response
PK	pharmacokinetics
ROC	receiver operating characteristic
UST	ustekinumab
TNF	tumor necrosis factor
iv	intravenous
sc	subcutaneous
SCR	sustained clinical response
IQR	interquartile range
